# Model of a DNA-Protein Complex of the Architectural Monomeric Protein MC1 from *Euryarchaea*

**DOI:** 10.1371/journal.pone.0088809

**Published:** 2014-02-18

**Authors:** Françoise Paquet, Olivier Delalande, Stephane Goffinont, Françoise Culard, Karine Loth, Ulysse Asseline, Bertrand Castaing, Celine Landon

**Affiliations:** 1 Centre de Biophysique Moléculaire, Centre National de la Recherche Scientifique UPR 4301, Université d'Orléans, Orleans, France; 2 Faculté des Sciences Pharmaceutiques et Biologiques, Institut de Génétique et Développement de Rennes, Centre National de la Recherche Scientifique UMR 6290, Université de Rennes1, Rennes, France; The University of North Carolina at Charlotte, United States of America

## Abstract

In *Archaea* the two major modes of DNA packaging are wrapping by histone proteins or bending by architectural non-histone proteins. To supplement our knowledge about the binding mode of the different DNA-bending proteins observed across the three domains of life, we present here the first model of a complex in which the monomeric Methanogen Chromosomal protein 1 (MC1) from *Euryarchaea* binds to the concave side of a strongly bent DNA. In laboratory growth conditions MC1 is the most abundant architectural protein present in *Methanosarcina thermophila* CHTI55. Like most proteins that strongly bend DNA, MC1 is known to bind in the minor groove. Interaction areas for MC1 and DNA were mapped by Nuclear Magnetic Resonance (NMR) data. The polarity of protein binding was determined using paramagnetic probes attached to the DNA. The first structural model of the DNA-MC1 complex we propose here was obtained by two complementary docking approaches and is in good agreement with the experimental data previously provided by electron microscopy and biochemistry. Residues essential to DNA-binding and -bending were highlighted and confirmed by site-directed mutagenesis. It was found that the Arg25 side-chain was essential to neutralize the negative charge of two phosphates that come very close in response to a dramatic curvature of the DNA.

## Introduction

The genomic DNA of all organisms across the three domains of life needs to be compacted and functionally organized. Wrapping-proteins, bending-proteins and bridging-proteins are thus involved and it appears that the underlying mechanisms are similar among *Bacteria*, *Archaea* and *Eukaryota* [1]. The two major modes of DNA packaging are 1) wrapping by histone proteins in *Eukaryota* (H2, H3 and H4 core histones) and *Archaea* (HMf histones), and 2) bending by architectural non-histone proteins in *Bacteria* (HU/IHF/Fis) and *Archaea* (Cren7/Sul7/MC1). DNA-bridging proteins have also been found in *Eukaryota* (H1linker histone), *Archaea* (Alba) and *Bacteria* (H-NS). Alba is the second most widely distributed archaeal chromatin protein after the archaeal histones [2], [3]. All *Crenarchaea* and *Euryarchaea* encode Alba with the exception of *Methanosarcina, Halobacteria* and *Thermoplasma* species [4]. Possibly not coincidentally, MC1 is present when Alba is absent. *Thermoplasma acidophilum*, another *Euryarchaea,* which is known to lack archaeal histones, encodes the HTa protein, a homolog of bacterial chromatin HU [5], [6].

MC1 is a member of the archaeal bending proteins and is the most abundant architectural protein present in *Methanosarcina thermophila* CHTI55 in laboratory growth conditions [7]. This small basic monomeric protein of 93 residues is structurally unrelated to other DNA-binding proteins [8], [9]. Its global fold consists of a pseudo barrel with an extension of the β-sheet (*β*4–*β*5) forming an arm (LP5). The secondary structure elements, namely an *α-*helix (Arg25-Arg34) and five *β-*strands *β*1 (Arg4–Arg9), *β*2 (Glu15–Gly21), *β*3 (Asp43–Glu49), *β*4 (Lys54–Asp66), and *β*5 (Lys78–Glu90), are all antiparallel and packed to each other. An antiparallel *β*-bulge composed of Val57, Glu87 and Arg88 is always present. The secondary structure elements are connected by five loops LP1 (Asp10–Asn14), LP2 (Lys22–Pro24), LP3 (Gly35–Pro42), LP4 (Arg50–Lys53), and LP5 (Ala67–Glu77).

MC1 protects DNA against thermal denaturation and shapes DNA by binding to it [7]. Its affinity for any double-stranded DNA is high (K_D_≈100 nM) and it recognizes and preferentially binds to bent DNA, such as four-way junctions (≈4-fold affinity) [10] and negatively supercoiled DNA minicircles (>10-fold affinity) [11]. More recently a SELEX (systematic evolution of ligands by exponential experiment) procedure revealed that it preferentially binds to a linear 15 base pair (bp) motif [AAAAACACAC(A/C)CCC(C/A)] with a particularly strong affinity (K_D_≈2 nM) [12], making it possible to investigate the DNA-binding mode of the protein by NMR spectroscopy. Visualization of DNA duplexes (176 bp) by electron microscopy revealed that the binding of MC1 induces sharp kinks so that the overall bend angle is estimated at 116° [13]. MC1 is too small to be directly visualized by electron microscopy but the relatively large segment of DNA (20–30 bp) that is protected from DNAse I suggests that DNA is wrapped around MC1 [14]. Hydroxyl radical footprinting together with a distamycin competition experiment demonstrated that the protein binds to DNA through its minor groove and that the binding site is composed of two areas of contact separated by approximately 10 bp [12]. Many other examples of architectural proteins that interact exclusively with the minor groove of DNA are found in the literature [15]: the TATA-binding protein (TBP), the male sex determining factor SRY, the lymphoid enhancer-binding factor 1 (LEF-1), the integration host factor (IHF) and the high mobility group I (HMGI). All of these minor groove-binding proteins bind with high affinity and varying degrees of sequence-specificity. Moreover, they exhibit very different global folds and use different strategies, or combinations of strategies, for recognition and binding.

We present here a new type of DNA-protein complex in which the monomeric protein MC1 binds on the concave side of the strongly bent DNA. Models were obtained by two successive and complementary docking approaches. The first one is based on a coarse-grained and interactive approach: a flexible and initially mildly-kinked double-strand DNA is driven into the electrostatic potential of the free static MC1 protein, thus producing a more kinked structure of the double-strand obtained upon protein interaction. The second step is flexible docking. It takes into account i) the 15 NMR models of the free MC1 protein to model the flexibility of the LP5 loop with regard to the core of the protein and ii) the 15 bp oligonucleotide generated in the first step and guided by the defined DNA-protein interface as ambiguous interaction restraints. Models of the DNA/MC1 complex are in good agreement with the previous experimental data provided by electron microscopy and biochemistry. Residues essential to the DNA-binding and -bending are highlighted by the structural complex and confirmed by site-directed mutagenesis.

## Materials and Methods

### DNA sequences

Only top strands are shown:

15 bp used for NMR and All Atom docking (**AAAAACACACACCCA**)

21 bp used for coarse grained docking (CTC**AAAAACACACACCCA**CGA)

26 bp used for EMSA (GCCTC**AAAAACACACACCCC**CGACAG).

### NMR sample preparation

The free protein NMR sample was prepared by concentrating ^15^N-MC1 to 1.6 mM (10 mM phosphate buffer pH 6, 100 mM NaCl, 1 mM EDTA, 10% D_2_O) as described previously [9].

Synthetic oligodeoxyribonucleotides (OliGold oligonucleotide quality) were purchased from Eurogentec (Liège, Belgium). The single-stranded oligodeoxyribonucleotides were characterized by NMR and annealed at a 1∶1 ratio. The free duplex concentration in the NMR sample was 2.5 mM (10% D_2_O).

The ^15^N-MC1/DNA complex was prepared by slowly adding the 7.5 mM DNA duplex solution (10 mM phosphate buffer pH 6, 100 mM NaCl, 1 mM EDTA, 10% D_2_O) to the 1.6 mM protein solution to give a final concentration of 1 mM.

The EDTA-C2-dT phosphoramidite was purchased from Eurogentec (Liège, Belgium). The EDTA labeled strands DNA1* and DNA2* were solid-phase synthesized, purified by RP-HPLC and characterized by ^1^H NMR. After annealing of the complementary strands, coordination of manganese was achieved by addition of 1.1 molar equivalent of MnCl_2_ to DNA1* or DNA2* solutions at pH 6. Chelex-100 beads (BioRad) were added to the DNA*Mn^2+^ solutions to remove metal excess. NMR samples for ^1^H_N_-Γ_2_ paramagnetic relaxation enhancement (PRE) measurements were prepared by mixing the purified protein ^15^N-MC1 and the DNA*Mn^2+^ at a molar ratio of 1∶1.2. The final complex concentration was 0.35 mM in 10 mM phosphate buffer pH 6, 100 mM NaCl and 10% D_2_O.

### NMR spectroscopy

All of the NMR experiments were performed on a 600 MHz Varian ^UNITY^INOVA spectrometer at 299 K. DSS (4,4-dimethyl-4-silapentane-1-sulfonic acid) was used as reference. Spectra were processed using NMRPIPE [16], and analyzed with NMRVIEW [17].

The ^15^N and ^1^H_N_ chemical shifts for free MC1 have already been reported [9] and those for bound MC1 were assigned from a combination of ^1^H-^15^N HSQC (heteronuclear single quantum coherence) and 2D ^15^N NOESY-HSQC (mixing time of 150 ms) spectra. Chemical shift perturbation (CSP) data of the protein were calculated and analyzed with SAMPLEX [18].

The ^1^H NMR resonances of the free 15-mer dsDNA were assigned from a combination of NOESY (nuclear Overhauser effect spectroscopy) (mixing times of 150 and 300 ms) and TOCSY (total correlation spectroscopy) (mixing time of 60 ms) spectra. The bound form was studied using NOESY (mixing times of 150 and 300 ms) and TOCSY (mixing time of 80 ms) spectra.

^1^H_N_-Γ_2_ values were determined as a difference in transverse relaxation rates (R_2_) for the paramagnetic and diamagnetic states of the bound protein. R_2_ for the diamagnetic state was given previously [9] and R_2_ for the paramagnetic state was measured with R_2_ relaxation delays of 2, 3, 4, 5, 6, 7, 8, 10, 12, 14, 16, 18, 20 and 24 ms. Volumes for the amide ^15^N-^1^H cross peaks were measured using NMRVIEW [17] and fitted with a single exponential decay function.

### Structure analysis

The Adaptive Poisson-Boltzmann Solver (APBS) program [19] was used within PyMOL to display the results of the calculations as an electrostatic potential molecular surface. The Platinum software [20] was used to calculate and visualize molecular hydrophobic/hydrophilic properties using the concept of “Molecular Hydrophobicity Potential” (MHP). Sequence homology and alignment were performed using online Protein BLAST software (blast.ncbi.nlm.nih.gov). The structures of nucleic acids were analysed using the CURVES+ software [21]. The figures were drawn with VMD [22], PyMOL [23] and MOLMOL [24].

### Docking of DNA onto the MC1 protein

Several steps were used to obtain DNA/MC1 complexes and are schematically reported in Supplementary Figure S1.

#### Coarse-grained docking with BIOSPRING

An interactive docking experiment was performed using the BioSpring program [25]. Flexible double-strand DNA was driven into the electrostatic potential of a static coarse-grained molecular shape of the free MC1 protein corresponding to the first model of the 2KHL PDB structures. DNA should be strongly curved in the final complex, as observed from electron microscopy experiments [13]. Taking this into account and in order to obtain a better convergence of the simulation, two kinked-DNA conformers were extracted from DNA-protein complexes available in the PDB (1A74, recognition DNA sequence of I-PpoI [26] and 1YTB, yeast TATA-box) and were used as starting structures. Given the 10 bp distance between the two contact regions that was observed experimentally, a 15 bp oligonucleotide would be too short to achieve an accurate docking procedure; a 21 bp oligonucleotide was therefore modeled. This coarse-grained ligand was simulated as a flexible double-stranded DNA following the protocol proposed in our previous study and using a 9 Å distance cutoff to form the elastic network [25]. The supplementary information that was used to interactively build the system and to place DNA in contact with the protein was as follows: i) the protein makes a double minor groove contact spaced by approximately ten base pairs in the double helix [12]; ii) Trp74 and Met75 are key residues to establish one of these contacts [27]; and iii) surface contact occurs near the T16 extremity (paramagnetic probe). We performed five replicas for each docking by considering each of the two different DNA structural patterns (1A74 and 1YTB).

#### High resolution model reconstruction of the DNA

All-atom 21 bp oligonucleotide models containing the specified sequence (**AAAAACACACACCCA**) and a high curvature were generated using the 3D-Dart server [28]. Ten most relevant all-atom 3D-Dart models were selected after superimposition (best RMSD of 3 Å) onto the low-resolution oligonucleotides from the DNA-MC1 complex generated by the BioSpring approach. The DNA-MC1 atomic models were obtained after shortening the 21 bp oligonucleotide to the 15 bp length used experimentally.

#### High resolution docking using HADDOCK

The default protein–DNA docking protocol described by van Dijk and Bonvin [29] which consists of i) rigid-body docking (1000 models), ii) a semi-flexible refinement stage (200 models), and iii) final refinement in explicit solvent (200 models), was used for all the docking runs using the HADDOCK web server [30]. The 15 models of the free protein were used to model the flexibility of the LP5 loop (2KHL.pdb). DNA structures were extracted from the 10 MC1-DNA models obtained by the interactive docking. Each DNA model was docked to the 15 free conformations of MC1 in solution. Planarity and base pairing restraints were used during all stages of the docking to preserve the helical conformation of DNA. Both protein and DNA were defined to be semi-flexible on all their length after the rigid-body docking stage. Ambiguous interaction restraints based on experimental information (CSPs, dynamics, paramagnetic probes, mutations…) were used: the side chain protons of Lys86, Arg88 and Ile89 were constrained to approach at least one proton belonging to A2A3A4A5C6:G25T26T27T28T29 with a distance of 5±1 Å. The side chain protons of Pro72, Trp74 and Met75 were constrained to approach at least one proton belonging to C13C14A15:T16G17G18 with a distance of 5±1 Å (Supplementary Figure S2).

### MC1 mutagenesis

Mutagenesis of MC1 was performed using standard protocols for site-directed mutagenesis with the plasmid pET24b-MC1 encoding for the MC1 protein. All of the mutants were verified by DNA sequencing of the entire gene. The proteins were expressed in BL-21(DE3) cells transformed with the appropriate plasmid and purified by three column chromatography steps (SP-Sepharose, Ultrogel AcA54, and Mono-S). The protein concentration was determined by absorption spectrophotometry, using the molecular absorbance coefficient of 11,000 M^−1^ cm^−1^ at 280 nm for wild-type and all mutants, except for the mutant Trp74Phe for which the molecular absorbance coefficient used was 5600 M^−1^ cm^−1^. Protein purity evaluated by SDS-PAGE was found to be better than 95 percent (Supplementary Figure S3). Correct molecular weights were observed by maldi-TOF mass spectrometry.

### Electrophoretic mobility-shift assays

The duplex DNA (26 bp) used for EMSA (electrophoretic mobility-shift assays) experiments comprises the consensus sequence that was previously determined by SELEX [12]. The single-stranded oligonucleotide (MWG-Eurofins) was first ^32^P-labeled at its 5′end then annealed to its complementary sequence in 10 mM Tris-HCl (pH 7.5), 1 mM EDTA, 150 mM NaCl by heating at 90°C for 3 min and slow cooling. EMSA reaction mixtures (10 µl) were prepared at 4°C by mixing DNA duplex and MC1 protein at concentrations indicated in the legend to each figure (Supplementary Figure S4), in binding buffer (10 mM Tris–HCl, 150 mM NaCl, 1 mM EDTA, 15 µg.ml^−1^ BSA, and 8% (v/v) glycerol, pH 7.5), followed by incubation for 15 min at 20°C. The different mixtures were loaded onto a polyacrylamide gel in TBE buffer (89 mM Tris-HCl, pH 8.3, 89 mM boric acid, 1 mM EDTA). Electrophoresis was run at 14 V/cm, for 1 hour (K_D_ measurement) or 3 hours (bending effect) at 20°C. After drying, gels (Supplementary Figure S4A) were quantified using a *β*-scanner (Typhoon Trio, Molecular Dynamics). The binding curves (Supplementary Figure S4B, C) were fitted to a single binding site model using the equation {Y  =  [MC1] / ([MC1] + K_D_app)} where Y is the fraction of bound DNA, [MC1] is the concentration of free protein, and K_D_app is the apparent dissociation constant. Under our experimental conditions, the free protein concentration is reasonably approximated by the total protein concentration.

## Results

### Determination of the DNA-binding surface of MC1

**Figure 1:**
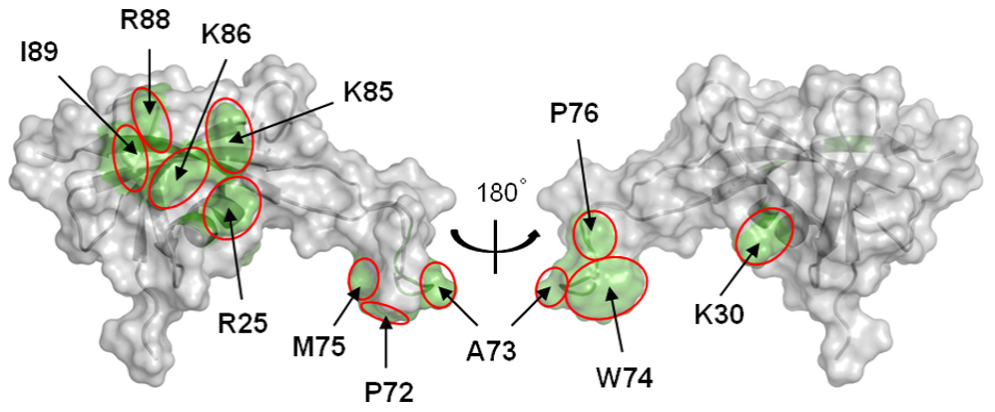
DNA-binding surface of MC1. The DNA-binding surface of MC1 is represented in green on the solvent-accessible surface. Residues which combine significant CSP and positive charge, or CSP and internal dynamics, are labeled.

As attested by the superimposition of ^1^H-^15^N HSQC spectra between the free and the DNA-bound form of MC1 (Supplementary Figure S5), the overall structure of the protein is largely unchanged upon binding. Measurement of ^1^H_N_ and ^15^N chemical shifts on both the free and bound forms of MC1 yielded the chemical shift perturbations (CSP) along the sequence of MC1. These CSP data were analyzed using the SAMPLEX software [18] and are reported in Supplementary Figure S5. Significantly perturbed residues were located in five sites: the α-Helix from Pro24 to Arg34, three residues in the center of the *β*3-strand (Ile45-Leu47), six residues belonging to the β4-strand (Phe58-Glu63), part of the loop LP5 (Pro72-Pro76) and practically all of the residues constituting the β5-strand (Val84-Glu90). It is worth noting that chemical shifts can report on both direct interaction and indirect effects such as remote conformational changes [31]–[33]. To refine our selection these CSP data were compared with the electrostatic potential of MC1 and with the flexible regions of MC1 defined by NMR relaxation data [9]. First, the CSP of residues located in the core of MC1 correlate with the basic patch constituted by the positive charges of Arg4, Lys22, Arg25, Lys30, Lys53, Lys54, His56, Arg71, Lys81, Lys85, Lys86, Arg88 and Lys91. Second, we have previously observed that Ile89 and the loop LP5 (67–77), mainly composed of hydrophobic residues (Ala67, Pro68, Pro72, Ala73, Trp74, Met75 and Pro76), possess considerable internal motions on the nanosecond time scale in the free protein and become much less mobile after binding with DNA [9], these residues have significant CSP too. In summary, we assume that residues of MC1, which present CSP after DNA-binding and belong to the basic surface or to the flexible regions, define the DNA-binding surface of MC1 (Figure 1). These residues belong to the α-helix (Arg25, Lys30), the LP5 arm (Pro72, Ala73, Trp74, Met75 and Pro76) and the β5-strand (Lys85, Lys86, Arg88 and Ile89).

### NMR features of the DNA after MC1-binding

#### Exchangeable imino protons

The hydrogen-bonded imino protons from the guanine and thymine nucleotides observed in the very low field region of the ^1^H NMR spectrum are highly sensitive indicators of the Watson-Crick base pairing and stability of DNA duplexes in solution. In the free 15 bp DNA, all of the thymine and guanine imino protons, with the exception of the terminal residues T16 and T30, were observable at 10°C indicating normal base stacking of a B-form for the duplex. In the bound DNA these imino protons plus T30 were observable at 25°C. MC1 stabilizes the duplex pairing of DNA as already observed for the DNA melting profiles with MC1 [7].

The largest CSP for the imino protons after binding were measured for G25 and T26 (>0.2 ppm), the imino proton of G25 is shielded and that of T26 is deshielded (Supplementary Figure S6A). This could be due to a local environment change such as a kink or an intercalation of amino acid side-chains between G25 and T26.

#### Nonexchangeable protons

The spectral region corresponding to the H1' (6.5–5 ppm), and H2, H6/H8 (8.4–7 ppm) DNA protons is quite free of protons belonging to the protein in the NOESY spectra of the DNA-MC1 complex (Supplementary Figure S7). Thus, it was possible to assign these protons except those belonging to G18, G19 and T20 due to overlapping. Their chemical shifts were compared to those of the free DNA and we observed that H1' of C6, C14 and T16 were particularly affected after binding (Supplementary Figure S6B). The H1' protons of the deoxyribose cycle point towards the minor groove; thus, the CSPs of H1' express perturbation in the minor groove. Moreover, the chemical environment of all the protons of the C6 nucleotide appears to be changed, supporting the hypothesis of a kink or an intercalation at the C6:G25 base pair level.

### Paramagnetic probes attached to nucleic acid

**Figure 2:**
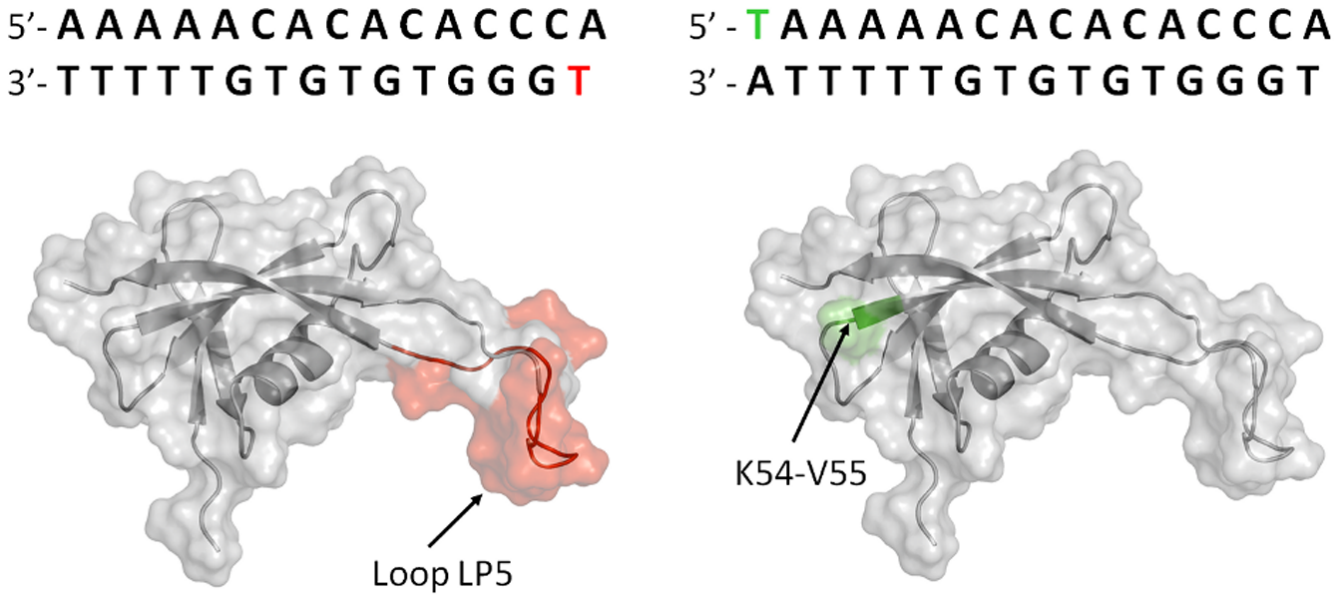
Relative orientation of the DNA extremity with respect to MC1. Sequence of the two oligonucleotides DNA1* (top) and DNA2* (bottom) including a paramagnetic probe; the color-coded **T** (red or green) represents dT-EDTA-Mn^2+^. The effect of each paramagnetic probe is shown on the three-dimensional surface of MC1 in red and green respectively.

Paramagnetic relaxation enhancement (PRE) of ^1^H-nuclei is a well-known source of qualitative and quantitative long-range distance information [34]. To determine qualitatively and rapidly the bound orientation of the protein relative to the DNA, we introduced a paramagnetic label (dT-EDTA-Mn^2+^) at the 5′-extremity of each DNA strand. The phosphoramidite for dT-EDTA is commercially available and can be incorporated at any chosen position using a DNA synthesizer. We synthesized two oligonucleotides containing a single specific binding site: the first labeled on the TG-rich strand at the 5′-terminal position (DNA1*) and the second labeled on the AC-rich strand by adding one thymine at the 5′-terminal position (DNA2*). ^1^H-^15^N HSQC spectra of MC1 in the two complexes DNA1*/MC1 and DNA2*/MC1 showed no chemical shift differences but broadened signals, attesting that spin labeling does not change the DNA-protein interaction or the protein structure in the complex. ^1^H_N_-Γ_2_ PRE is defined as the difference in the transverse relaxation rates of the paramagnetic (after adding MnCl_2_) and diamagnetic states. ^1^H_N_-Γ_2_ PREs were measured for the backbone amide groups (^1^H_N_). Large magnitude ^1^H_N_-Γ_2_ PREs were observed only for the regions close to the dT-EDTA-Mn^2+^ probes. A significant relaxation enhancement was observed for the residues Lys69-Lys81 belonging to the same face of the arm LP5 and located in the regions closest to the dT-EDTA-Mn^2+^ site of the DNA1*Mn^2+^/MC1 complex, whereas the residues Lys54 and Val55 close to the dT-EDTA-Mn^2+^ site of the DNA2*Mn^2+^/MC1 complex exhibited ^1^H_N_-Γ_2_ values that were slightly higher (Figure 2 and supplementary Figure S8). These data immediately provided qualitative information about the orientation of the protein on the DNA duplex. Residues Lys69-Lys81 belonging to the arm LP5 are undeniably close to T16 and residues Lys54-Val55 are close to the 5′-supplementary thymine added before A1.

### Molecular models of the DNA-MC1 complex

**Figure 3:**
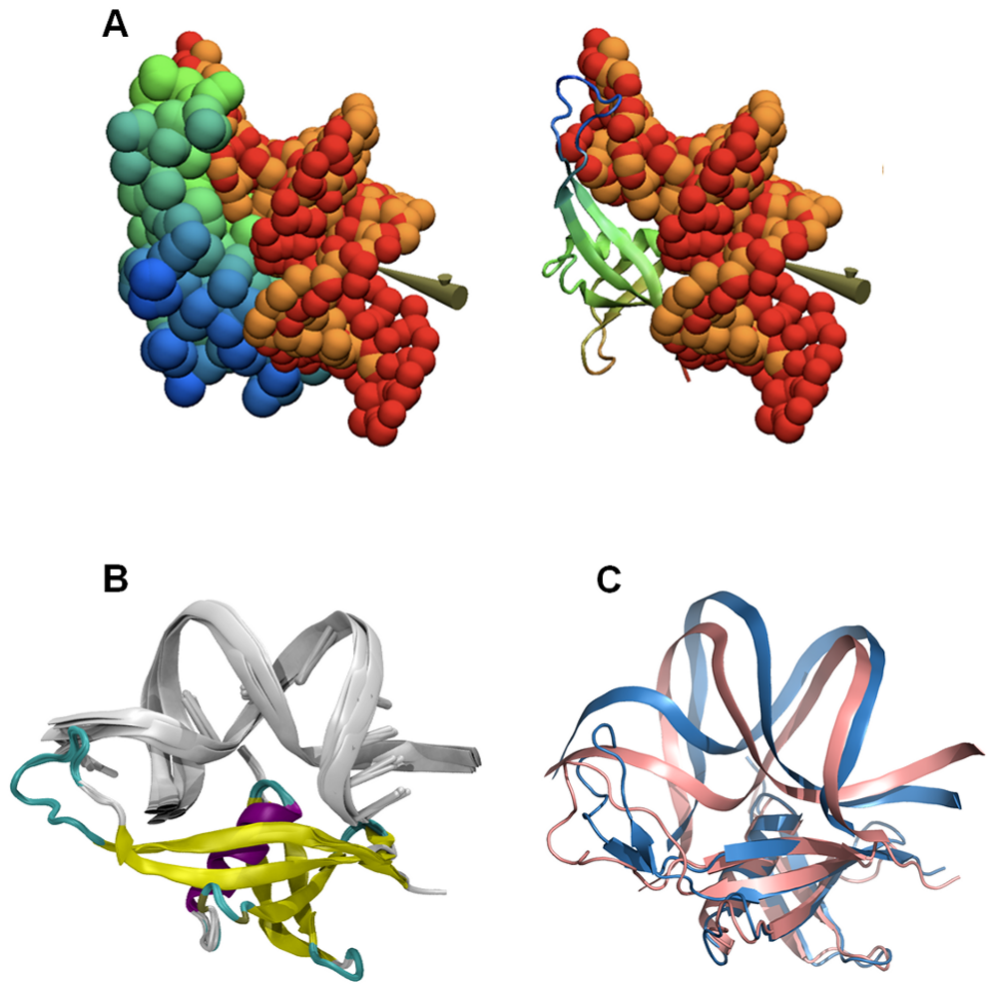
Proposed 3D model for MC1 bound to DNA. (A) Superimposition of two positions for double-strand DNA (orange and red spheres) after docking on the MC1 static molecular shape (green-blue, cartoon or spheres) by interactive driving of the flexible ligand through electrostatic potential of the protein, in accordance with the experimental data. (B) Ten final models for the DNA-MC1 complex (silver cartoon and colored cartoon respectively) proposed after all-atom reconstruction. Average DNA axis curvature measured by the Curves program on these models is 109±6°. (C) Superimposition of 2 of the 8 models of DNA-MC1 complex resulting from HADDOCK. The DNA angle curvature seems to be correlated to the position of the flexible protein LP5 loop: 122° for model 1 (blue) and 81° for model 8 (pink).

We performed a preliminary interactive docking experiment at low-resolution by simulating the association of a static molecular shape for MC1 with a flexible augmented elastic network model (aENM) for the oligonucleotide. All ten runs - i.e. driving of DNA double-strand through the electrostatic grid - strongly converged to highly reproducible DNA positions with an RMSD of 2 Å found on the phosphate groups (Figure 3A). This RMSD value is reasonable for low-resolution simulations. From these coarse-grained structures, all-atom reconstruction was performed to propose models of the DNA-MC1 complex (Figure 3B).

**Figure 4:**
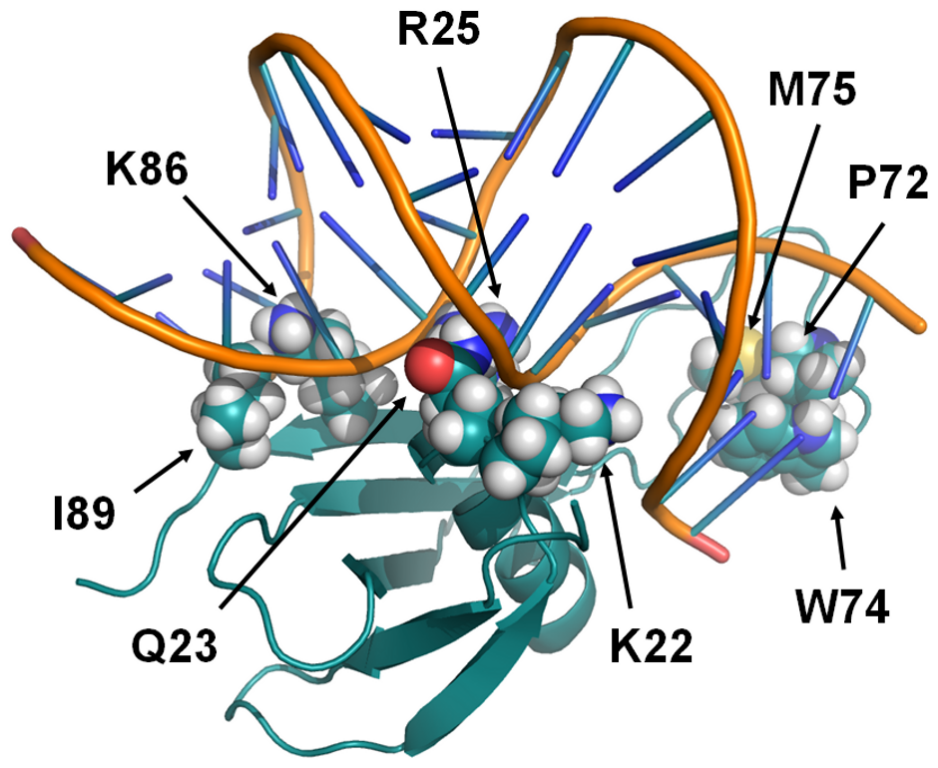
Model of MC1 interaction with the bent 15 bp DNA. The side chains of K22, P72, W74, M75, K86 and I89 contact the minor groove of DNA and that of R25, with the possible help of Q23, neutralizes the negative phosphates in the shrunk major groove.

The average DNA curvature obtained from the 10 models is about 109 ± 6°, which is a much greater angle than that of the starting DNA conformers (1A74: 49.3° and 1YTB: 71.4°) and in agreement with electron microscopy results [13]. The analysis of these models highlights two areas of contact in the minor groove located at the extremities of the 15 bp DNA. The first area is located in the β5-strand around Lys85, Lys86, Arg88 and Ile89 contacting the A-tract. The second area is part of the loop LP5 around Asn70, Arg71, Pro72, Trp74 and Met75 which contacts T16, G17 and G18. Strong additional electrostatic contacts involve A5C6 and the T20G21 phosphate backbone with Arg25 side-chains. These predicted models were in agreement with the experimental DNA-binding surface of MC1 defined above (Figure 1). However, the docking was performed with only one rigid protein model for MC1 and was not representative of the flexibility of the LP5 loop with regard to the core of the protein. We therefore performed a second docking with the commonly used HADDOCK program using the 15 free conformations of MC1 in solution and 10 15 bp DNA models extracted from the previous docking. Both the protein and DNA were considered semi-flexible molecules. Eight models of the DNA-MC1 complex were selected from the more populated clusters (∼140/200 pdb). Their bound DNA is bent with an average curvature of 104±21° (Supplementary Figure S9). The angle of curvature depends on the position of the loop LP5 with regard to the core of MC1. As in the first models, the protein hangs on to the extremities of the DNA by inserting the expected residues Pro72, Trp74, Met75, Lys85, Lys86, Arg88 and Ile89 in the minor groove. The side chain of Lys22 is most probably involved in the minor groove interaction as well (Figure 4). As a consequence of this dramatic curvature, the A5pC6 and T20pG21 phosphates move closer to each other and the side chains of Arg25 and Gln23, which were not constrained by our ambiguous interaction restraints (AIRs) during the docking, are positioned in the major groove (Figure 4). In the center of the 15 bp oligonucleotide (CACACA region) the minor groove is widened and shallow whereas the major groove is narrow and deep (Supplementary Figure S9).

### Functional validation of the DNA-MC1 model

**Figure 5:**
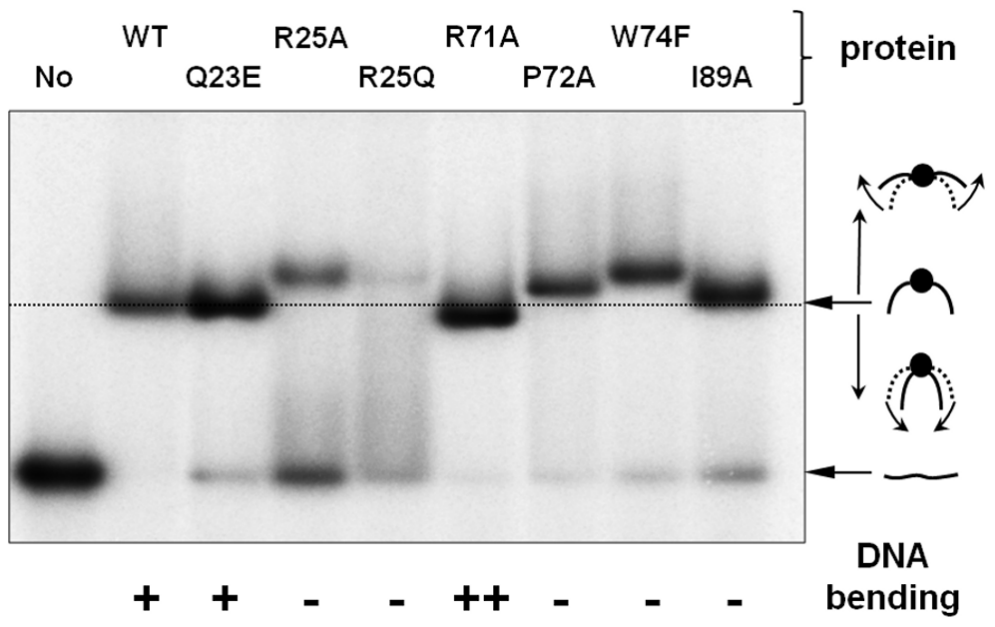
DNA bending ability of the WT and mutant MC1 proteins evaluated by EMSA. EMSA experiment was performed as described for K_D_app measurement: 0.1 nM of 5′-[^32^P]-labeled 26 bp DNA were incubated with the WT or mutant MC1 protein (at 20 nM final concentration, excepting R25A and R25Q mutant versions for which 100 nM was used). At equilibrium, assays were analyzed by EMSA as described in the *Materials & Methods* section. After 3 hours of electrophoresis, the gel was dried and visualized by autoradiography. The relative electrophoretic mobility of the protein/DNA complex provides an evaluation of the bending ability of the MC1 version considered. With this short DNA duplex, an apparent greater mobility of the nucleoprotein complex is expected for a protein with a greater DNA bending.

Directed mutagenesis was conducted to determine whether amino acids predicted in our models to be in areas of contact with DNA are required for DNA binding. In a previous study, the DNA-binding affinity of Trp61, Trp74 and Met75 mutants was compared with that of the wild-type protein [27]. We showed that the two residues located in the LP5 arm are likely involved in the interaction since substituting Trp74 for Phe and Met75 for Leu led to a decrease in the DNA-binding affinity. On the other hand, and in accordance with our current model, Trp61 located in the β4-strand is probably not directly involved in DNA binding since its substitution for Phe had no effect. In the same study, we also showed that Trp74 and Met75 are involved in DNA bending since their substitution by Ala induced a loss of the capacity of the protein to recognize bent DNA as well as a strong reduction in the protein's ability for DNA bending. Finally, we observed that the substitution Pro76Ala does not affect DNA binding (unpublished results). To complete this analysis, we constructed a new series of MC1 mutants. Altogether, eight proteins were compared for their ability to bind a 26 bp DNA containing the consensus sequence used in the NMR experiments. EMSA were used to separate bound from free DNA (Figure 5 and Supplementary Figure S4) and to determine K_D_ values for each mutant (Table 1). The DNA affinities of the mutants are clearly in accordance with our model of DNA-MC1 binding, implying three area sites. First, we attest that LP5 is involved in DNA binding: substituting Arg71 or Pro72 for Ala induced a slight decrease in the affinity (≈3-fold) whereas the conservative substitution Trp74Phe induced a larger effect (7-fold). A second area involves Ile89 located in the β5-strand, whose substitution for Ala greatly affected the interaction (12.8-fold affinity). Finally, the third point of DNA-MC1 contact was validated by substitutions of Gln23 and Arg25 located in the α-helix. The replacement of Gln23 by a Glu distinctly decreased the binding (7.5-fold affinity), and the effect was larger still with Arg25, whose replacement by Ala or Gln greatly affected the binding (>100-fold affinity). According to the data in the literature, the loss of an electrostatic interaction has a greater effect on DNA binding than the loss of a hydrophobic bond [35]. Mutation of Arg25 strongly affected the binding, which further argues that Arg25 is involved in an electrostatic contact with the DNA backbone.

**Table 1:** Apparent dissociation constant (K_D_app) for the WT and mutant MC1 proteins obtained from EMSA experiments.

MC1	WT	Q23E	R25A	R25Q	R71A	P72A	W74F	I89A
**K_D_app (nM)**	1.2±0.1	9.05±2	**161±14**	**552±34**	3.6±0.2	4.2±0.5	8.4±1	15.4±2
**DNA Bending**	+	+	−	−	++	−	−	−

Binding curves (Supplementary Figure S3) obtained from EMSA experiments were fitted with the equation: Y  =  [MC1] / ([MC1] + K_D_app), where Y is the fraction of the bound 26 bp DNA, and [MC1] is the free protein concentration approximated by the total protein concentration in our experimental conditions. Each K_D_app value is the mean ± standard deviation of three independent experiments.

EMSA also provided evidence for DNA bending. We previously observed an expected behavior with short DNA (<30 bp): a complex in which DNA is not or is only slightly bent migrates less rapidly than a complex in which DNA is tightly bent [27], [36]. Clearly, complexes with the mutants Arg25Ala/Gln, Pro72Ala, Trp74Phe and Ile89Ala migrate less rapidly than complexes with the WT protein (Figure 5): these four residues are therefore likely to be involved in DNA bending. On the contrary, Arg71Ala forms a complex that migrates slightly faster than WT MC1 and the Gln23Ala protein has no visible effect on the mobility of the complex. The trend in the mutant capacity to bend DNA is: Arg71Ala > WT ≈ Gln23Ala > Ile89Ala > Pro72Ala > Trp74Phe ≈ Arg25Ala/Gln.

## Discussion

**Figure 6:**
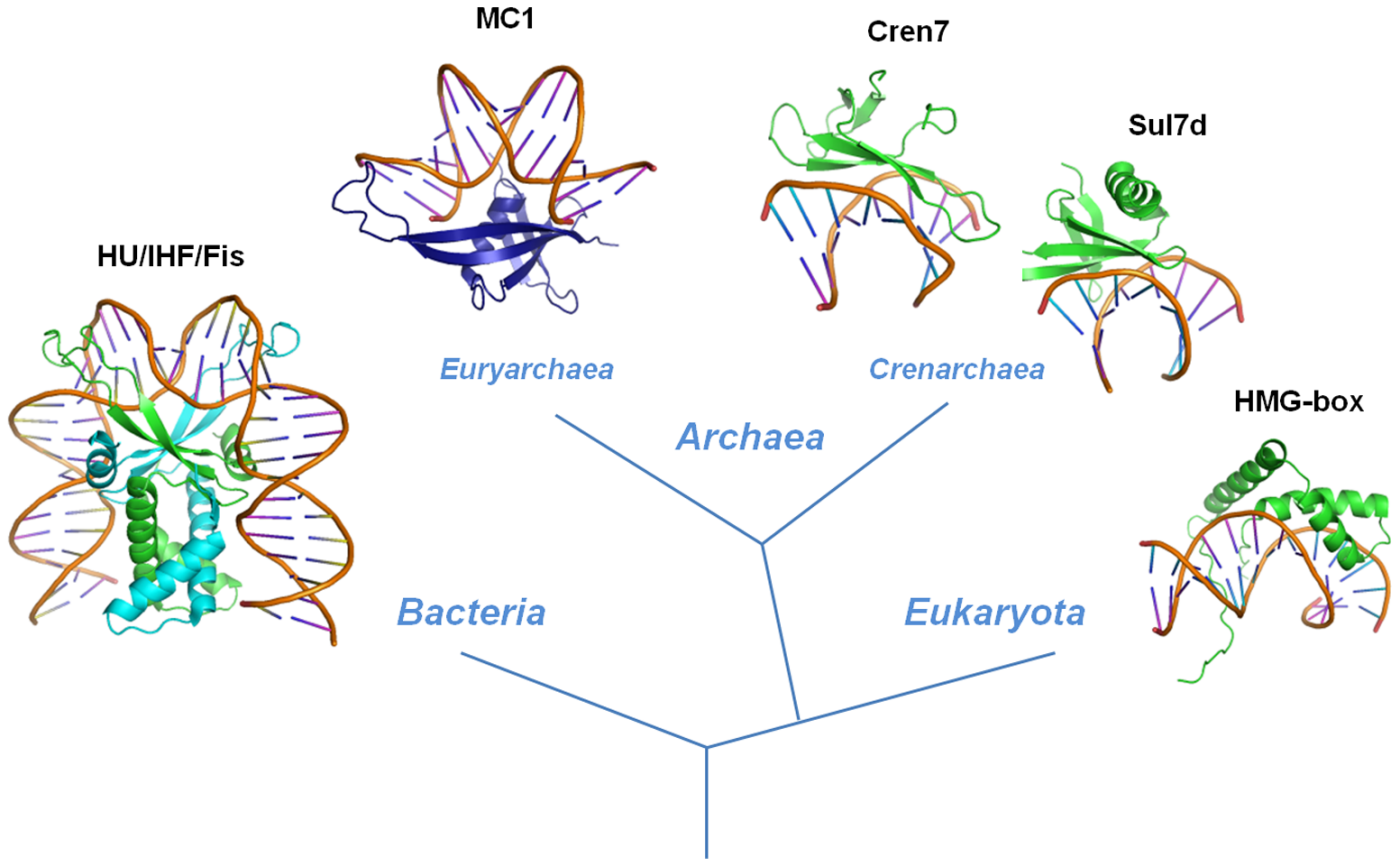
Representative 3D-structures of complexes with DNA-bending proteins among the three domains of life. The dimeric bacterial protein IHF (PDB 1IHF) and the monomeric *Euryarchaeal* protein MC1 contact the concave side of the DNA curvature. In *Eukaryota* and *Crenarchaea* subdomain the proteins SRY (HMG-box protein) (PDB 1J46), Sul7d (PDB 1AZP) and Cren7 (PDB 3KXT) contact the convex side of the DNA curvature.

Our experimental results converge to a protein/DNA model, in which the monomeric protein MC1 interacts on the concave side of a strongly bent DNA: 1) MC1 bears structural similarities to the small basic architectural proteins Sul7d and Cren7, belonging to the *Sulfolobus* strains of the *Crenarchaea* subdomain [37], [38], and interacts with the DNA minor groove. However, the later proteins bind on the convex side of the DNA curvature (Figure 6); and 2) Protein interactions with the concave side of DNA curvature have only been observed so far for dimeric proteins, such as histone-like HU or IHF, bound to U-shape DNA [39] (Figure 6). DNA-MC1 recognition is probably based on the shape readout such as minor groove narrowing, kink and bending [40]. Slight differences in minor groove shape, leading to slight differences in electrostatic potential may allow a fine grained recognition [41].

### Specific minor groove interactions

#### Minor groove narrowing in the A-tract

A-tracts are known to rigidify the double-strand helix of DNA [42] and have a bending propensity toward their narrow minor grooves [43]. The binding of arginine residues to the narrow minor groove is a widely used mode for DNA-protein recognition [44], [45]. The narrow minor groove of MC1 induced by the A1-A5 tract is recognized by the basic residues Lys85, Lys86 and Arg88. In their proximity, the great flexibility of the bulge (Val57, Glu87, Arg88) [9] is probably essential for the specific positioning of the Ile89 side-chain in the A-tract minor groove. Site-directed mutagenesis of MC1 coupled to binding experiments (Figure 5 and Table 1) showed that the side-chain of Ile89 is important for binding and contributes to the curvature of DNA. Some architectural proteins are known to use additional intercalating residues in the AT rich minor groove [15] such as the Phe-Met dipeptide for SOX17 protein [46] and Pro for IHF (HU) dimer [47].

#### Flexible CpA steps

Among the 10 possible dinucleotides, CpA, TpA and TpG are the most flexible steps, as they are weakly stabilized through base stacking interactions [40]. The MC1 sequence contains three consecutive CpA steps, giving the 15 bp sequence great flexibility. Moreover, the cytosine C6 is adjacent to the A-tract and, with this specific sequence, it was reported that bending and kinking were able to enhance each other [48]. This great flexibility is certainly responsible for the dramatic bending induced by MC1. Hence, the presence of the positive side-chain of Arg25 is essential to neutralize the repulsive negative charges of the phosphates belonging to the closely spaced A5pC6 and T20pG21 steps.

#### Minor groove widening in the CCCA sequence

The hydrophobic side-chains of Pro72, Trp74 and Met75 residues located in the mobile arm of MC1 are well positioned to bind the widened minor groove composed of the CCC sequence with possible intercalation in the terminal CpA step. Differences in the hydrophobicity of the mutated proteins could explain the observed differences in DNA bending (Supplementary Figure S10). Clearly, the mutation of Arg71 into Ala enhances the hydrophobicity of the LP5 arm, which may explain the strong DNA curvature observed for the Arg71Ala mutant. Indeed, hydrophobic contacts with bases are used by many architectural proteins such as TBP, SRY and LEF-1 that only contact the minor groove. Recently [49], a structural study of the CCAAT-binding complex revealed a very shallow and widened minor groove around the CCA steps with a DNA kink stabilized by the intercalation of a phenylalanine side-chain at the CpA step.

### DNA-recognition by the Euryarchaeal MC1 family

**Figure 7:**
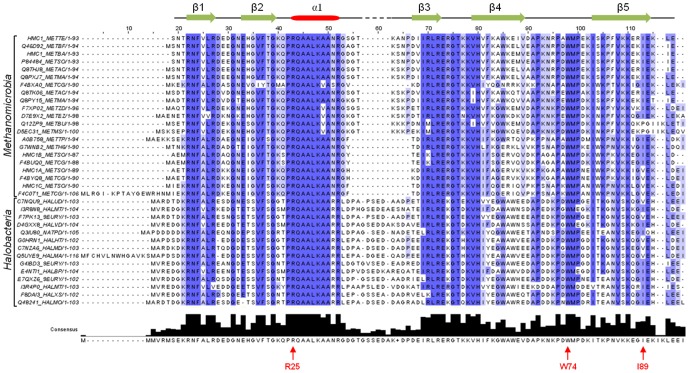
Sequence alignment of MC1 from *Methanosarcina thermophila* (HMC1_METTE) with other MC1 from the *Methanomicrobia* and *Halobacteria* classes. Secondary-structure elements of MC1(HMC1_METTE) are given above the sequences. Key residues indicated under the consensus sequence respect the numbering of the MC1 (HMC1_METTE) sequence.

Three regions of MC1 are essential to the DNA-recognition: the first is composed of the basic amino acids Lys85, Lys86, Arg88 and the isoleucine Ile89 located on the triple-stranded β-sheet; the second region involves the hydrophobic residues Pro72, Trp74 and Met75 in the arm; and to connect these two regions the Arg25 residue located on the first turn of the α-helix. These residues, except for Lys85, Arg88 and Ile89, are conserved among the other MC1 proteins from *Methanomicrobia* and *Halobacteria*, which are two classes of *Euryarchaea* (Figure 7). Arg88 is often replaced by Gly or Lys, which are other residues capable of contacting the narrow minor groove [50], [51], and Ile89 is sometimes substituted with Val, positioning a Γ-methyl group in the same way. An important point to note is the complete conservation of the residues Pro24 to Lys30, belonging to the α-helix, which seems to ensure the correct positioning of the Arg25 side-chain. In our opinion, the proposed model built for *Methanosarcina thermophila* MC1 is representative of MC1 from *Methanomicrobia* and *Halobacteria* species and more generally from the *Euryarchaeal* subdomain (Figure 7).

Bending proteins, used by all organisms across the three domains of life to compact genomic DNA, belong to distinct families of small, basic and abundant chromatin proteins (Figure 6). Many 3D structures of DNA complexes with bending proteins are deposited in the Protein Data Bank and most of them were obtained by crystallography: In *Bacteria*, 5 structures are known with HU [52]-[54], 11 with IHF [52]-[57], and 12 with Fis [58]. The dimeric proteins contact the concave side of DNA through three recognition sites in the minor groove. In *Eukaryota*, many tridimensional structures of HMG box-DNA complexes have been determined [46], [59]. The monomeric HMG proteins wrap around the DNA by minor groove recognition. In *Archaea*, only tridimensional structures of DNA-protein complexes belonging to the *Crenarchaea* subdomain are known: 6 structures with Sul7d (Sso7d/Sac7d) [60]–[62], and 3 structures with Cren7 [38], [63]. These monomeric archaeal proteins, like HMG proteins, contact the convex side of the DNA curvature. To our knowledge, the known solution structures are obtained only for proteins wrapping around DNA, such as HMG [64]–[67] and Sul7d [60]–[62], with many intermolecular NOEs between the protein and DNA. For the DNA-MC1 complex, the recognition type is completely different, in that the monomeric protein contacts the concave side of the DNA curvature.

In summary, the DNA-MC1 binding site is composed of two areas of contact in the minor groove. First, the narrow minor groove induced by the A1-A5 tract is recognized by the basic residues Lys85, Lys86 and Arg88 of MC1. In their proximity, the great flexibility of the bulge (Val57, Glu87, Arg88) seems to be important for the specific positioning of the Ile89 side-chain in the A-tract minor groove. Second, the hydrophobic side-chains of Pro72, Trp74 and Met75 residues located in the mobile arm of MC1 are well positioned to bind the minor groove composed of the CCCA sequence, with possible intercalation in the terminal CpA step. Then, three central and flexible CpA steps adjacent to the A-tract are responsible for the dramatic bending of DNA. The presence of the positive side-chain of Arg25 is essential to neutralize the repulsive negative charges of the phosphates in the major groove. To our knowledge, this is the first description of the interaction of a monomeric protein that takes place on the minor groove and on the concave side of the DNA curvature. This atypical DNA-MC1 structural model adds to our knowledge on DNA condensation observed across the three domains of life.

## Supporting Information

Figure S1 **Protocol used for the modeling of DNA-MC1 complexes.** The three successive steps used are from left to right: i) docking using BIOSPRING which gives coarse-grained models of the complex, ii) high-resolution model reconstruction of DNA with the specified sequence, and iii) high-resolution docking using HADDOCK which gives all-atom models of the complexes.
(TIF)

Figure S2 **Ambiguous Interaction Restraints used in HADDOCK runs.** The side-chain protons of Lys86, Arg88 and Ile89 are constrained to approach at least one proton belonging to A_2_A_3_A_4_A_5_C_6_:G_25_T_26_T_27_T_28_T_29_ with a distance of 5±1 Å (green rectangle). The side chain protons of Pro72, Trp74 and Met75 are constrained to approach at least one proton belonging to C_13_C_14_A_15_:T_16_G_17_G_18_ with a distance of 5±1 Å (red rectangle).
(TIF)

Figure S3 **SDS-PAGE analysis of purified proteins.** 2 µg of each protein (MC1 WT and mutants as indicated) were loaded on an EZ-run™ gel (Fischer Scientific). Staining with InstantBlue™ Coomassie followed by quantification using ImageQuant software showed purity higher than 95%.
(TIF)

Figure S4 **Comparative DNA binding properties of the WT and mutant MC1 proteins.** 5′-[^32^P]-26 bp DNA duplex (0.1 nM) containing the MC1 15 bp high affinity sequence was incubated with increased concentrations of WT and mutants versions of MC1 (0.05–10 nM protein range). At equilibrium, assays were analyzed by EMSA under conditions defined in the *Materials & Methods* section. (A) Example of a gel autoradiography obtained with the WT MC1 protein. (B) and (C) After autoradiography, the bands corresponding to the free and bound DNA probe were quantified and each point of titration experiments represents the mean value obtained for three independent experiments. Apparent dissociation constants (K_D_) were extracted from these curves by fitting to a single binding site using the equation: Y  =  [MC1] / ([MC1] + K_D_) and reported in table 1.
(TIF)

Figure S5 **Analysis of MC1 CSP upon DNA-binding.** (A) Superimposition of the ^15^N HSQC spectra of the free protein (blue) and the bound protein (red); H^N^ and N chemical shifts as a function of free MC1 chemical shifts. A line was added to highlight the linear trend. (B) CSP values as a function of residue number. (C) Analysis of CSP with SAMPLEX: the MC1 protein backbone is shown as a cartoon; colored regions correspond to residues showing no (grey), medium (orange) or strong (red) chemical shift variations upon complexation of oligonucleotide to protein as recorded in NMR experiments; the five loops and key residues are labeled and the MC1 sequence with CSP is reported. Residues are located in five sites: Pro24-Arg34 (α-Helix), Ile45-Leu47 (β3-strand), Phe58-Glu63 (β4-strand), Pro72-Pro76 (loop LP5) and Val 84-Glu90 (β5-strand).
(TIF)

Figure S6 **CSP analysis of DNA resonances upon MC1-binding.** (A) CSP of the exchangeable protons upon MC1-binding: the bound amino protons (NHb), the free amino protons (NHf) and the imino protons (NH) are indicators of the Watson-Crick base pairing. (B) CSP of the nonexchangeable protons after MC1-binding. The anomeric H1' protons of deoxyribose and the aromatic H2 protons of adenine are located in the DNA minor groove. The H6 protons of cytosine/thymine, the H8 protons of adenine/guanine, the H5 protons of cytosine and the methyl group of thymine are located in the DNA major groove.
(TIF)

Figure S7 **Assignment of the 15 bp DNA protons after binding to MC1.** The NOESY spectrum was recorded on the complex (1 mM) in 10 mM phosphate buffer pH 6, 100 mM NaCl, 1 mM EDTA, 10% D_2_O at 26°C with a mixing time of 120 ms on a 600 MHz spectrometer.
(TIF)

Figure S8 **Intermolecular PREs for the MC1/DNA complex.** Intermolecular PRE ^1^H_N_-Γ_2_ profiles obtained for each EDTA-Mn^2+^ DNA.
(TIF)

Figure S9 **DNA groove geometry in the complex.** The 8 models of 15 bp DNA bound to MC1 were analyzed using Curves+: (A) Schematic view of the bound DNA in the model 1 of the complex; the global helical axis is represented in blue and the backbone spines in red. (B) The DNA axis total bend, calculated for each model by Curves+, corresponds to the cumulative axis bend all along the oligonucleotide. (C) Groove parameters (width and depth) were calculated for each model.
(TIF)

Figure S10 **Three-dimensional representation of the surface potentials of the WT MC1 protein and its different mutants.** Positive and negative electrostatic potential isosurfaces are respectively shown in blue (+250 kTe) and red (−15 kTe). Hydrophobicity/hydrophilicity is mapped on black/light grey molecular surfaces. Each mutation is located on the molecular surface of MC1with an arrow.
(TIF)
