# Supplementary figures and images for: Model of a DNA-Protein Complex of the Architectural Monomeric Protein MC1 from Euryarchaea
Source: PLoS One. 2014 Feb 18;9(2):e88809. doi: 10.1371/journal.pone.0088809 (PMC3928310; doi:10.1371/journal.pone.0088809)

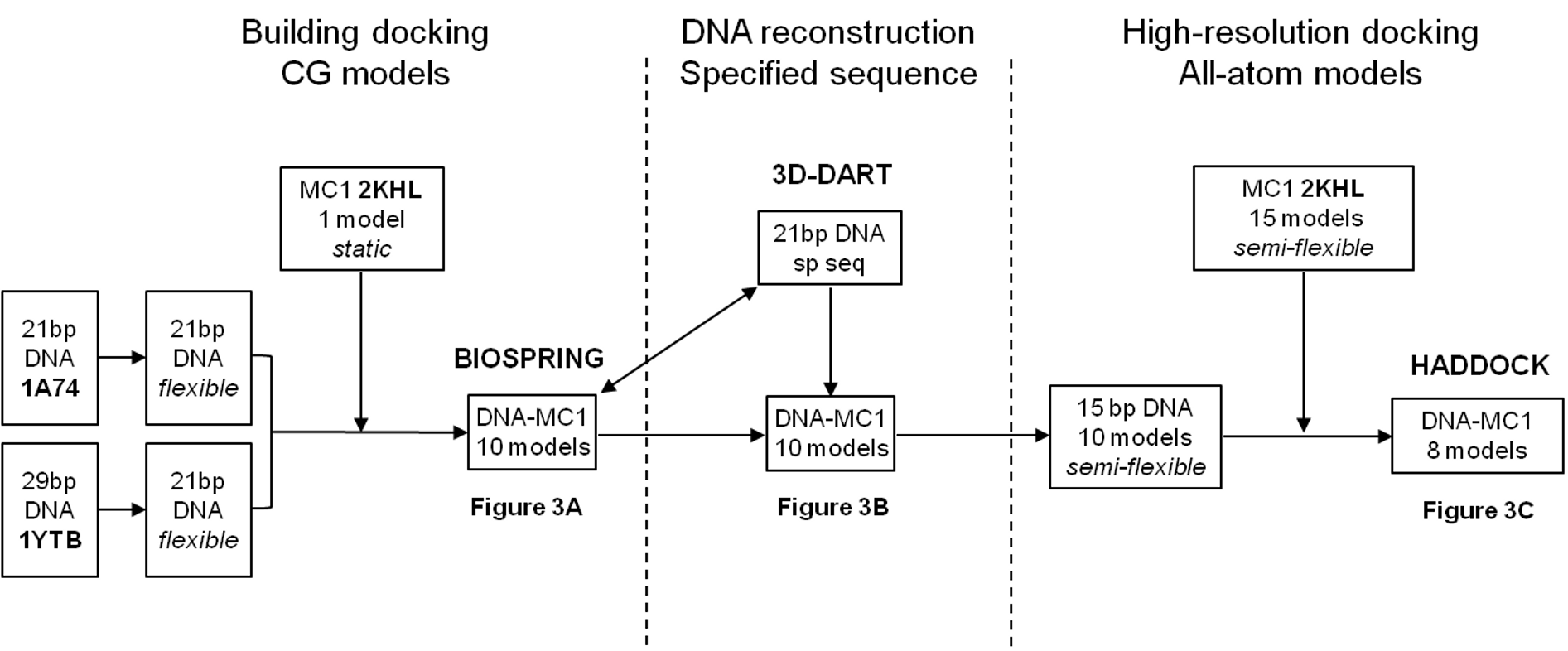

Supplement: Figure S1 — Protocol used for the modeling of DNA-MC1 complexes. The three successive steps used are from left to right: i) docking using BIOSPRING which gives coarse-grained models of the complex, ii) high-resolution model reconstruction of DNA with the specified sequence, and iii) high-resolution docking using HADDOCK which gives all-atom models of the complexes. (TIF) [file pone.0088809.s001.tif]

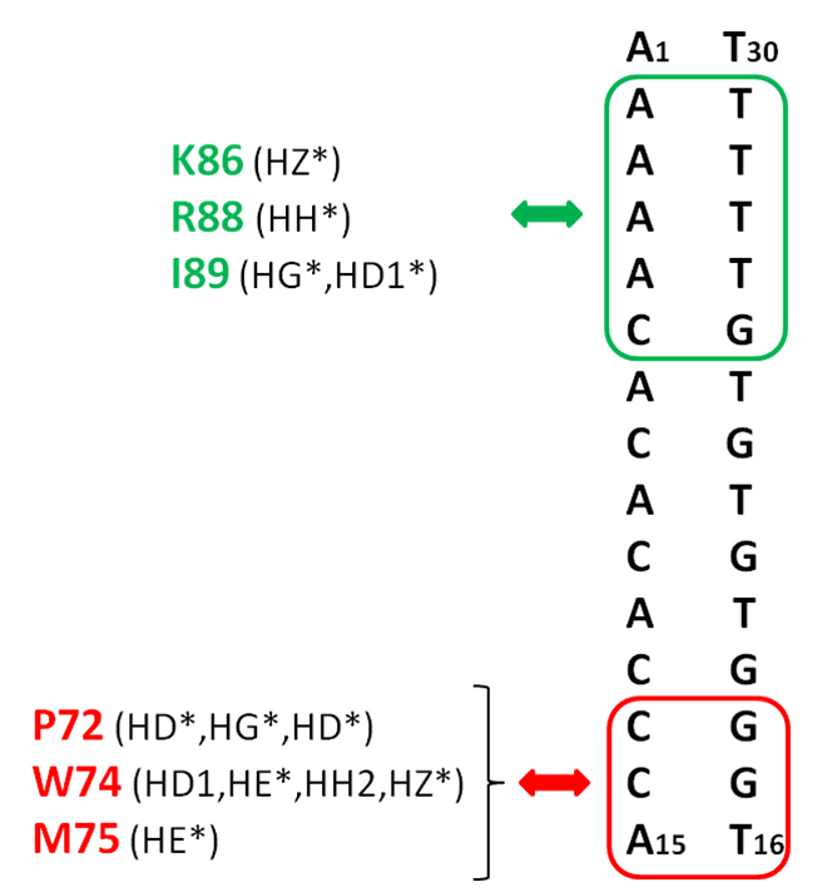

Supplement: Figure S2 — Ambiguous Interaction Restraints used in HADDOCK runs. The side-chain protons of Lys86, Arg88 and Ile89 are constrained to approach at least one proton belonging to A2A3A4A5C6:G25T26T27T28T29 with a distance of 5±1 Å (green rectangle). The side chain protons of Pro72, Trp74 and Met75 are constrained to approach at least one proton belonging to C13C14A15:T16G17G18 with a distance of 5±1 Å (red rectangle). (TIF) [file pone.0088809.s002.tif]

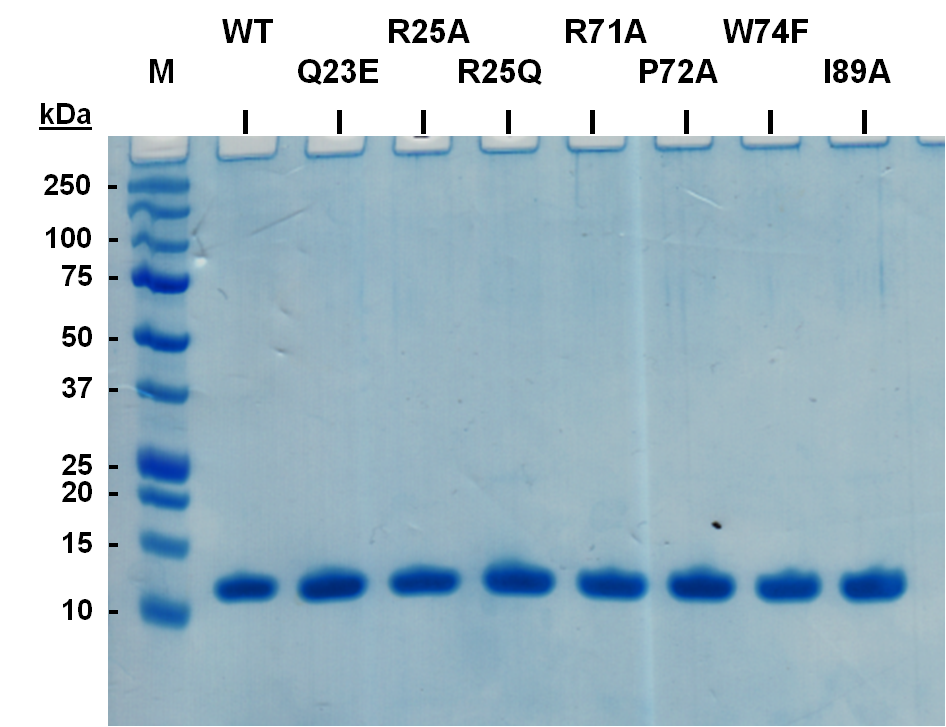

Supplement: Figure S3 — SDS-PAGE analysis of purified proteins. 2 µg of each protein (MC1 WT and mutants as indicated) were loaded on an EZ-run™ gel (Fischer Scientific). Staining with InstantBlue™ Coomassie followed by quantification using ImageQuant software showed purity higher than 95%. (TIF) [file pone.0088809.s003.tif]

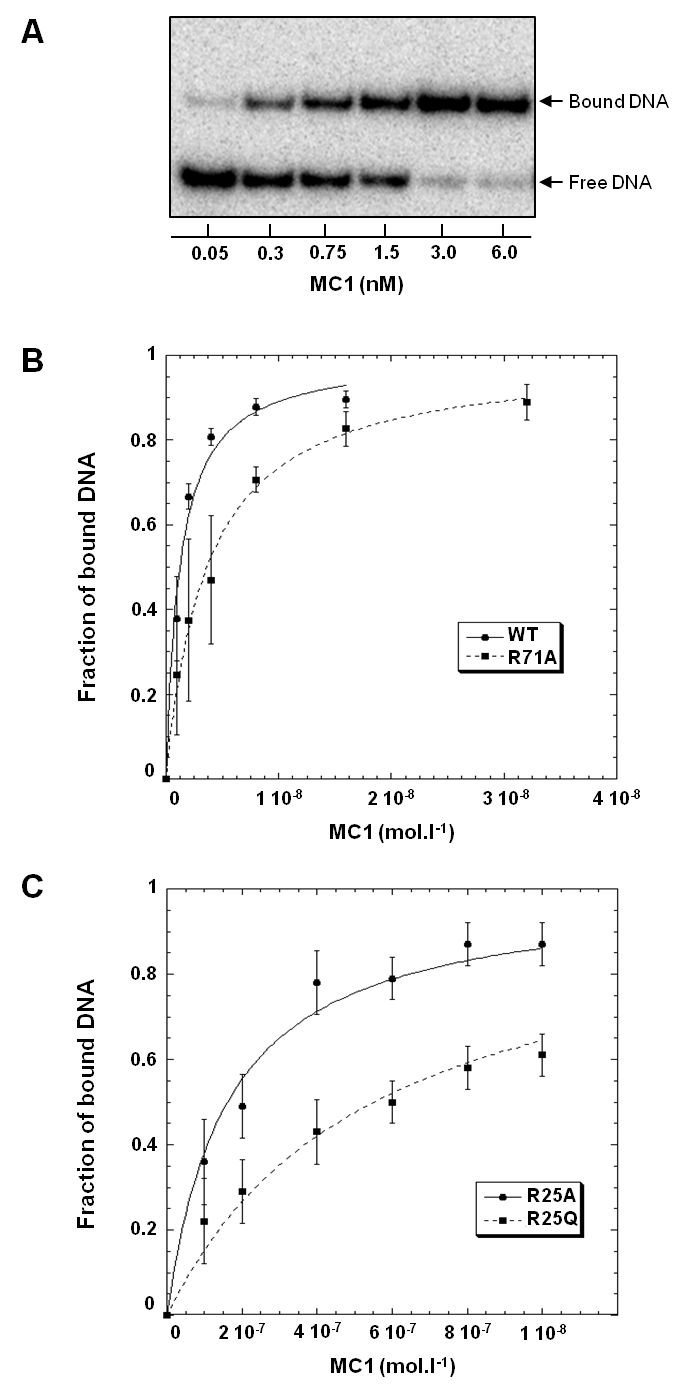

Supplement: Figure S4 — Comparative DNA binding properties of the WT and mutant MC1 proteins. 5′-[32P]-26 bp DNA duplex (0.1 nM) containing the MC1 15 bp high affinity sequence was incubated with increased concentrations of WT and mutants versions of MC1 (0.05–10 nM protein range). At equilibrium, assays were analyzed by EMSA under conditions defined in the Materials & Methods section. (A) Example of a gel autoradiography obtained with the WT MC1 protein. (B) and (C) After autoradiography, the bands corresponding to the free and bound DNA probe were quantified and each point of titration experiments represents the mean value obtained for three independent experiments. Apparent dissociation constants (KD) were extracted from these curves by fitting to a single binding site using the equation: Y = [MC1] / ([MC1] + KD) and reported in table 1. (TIF) [file pone.0088809.s004.tif]

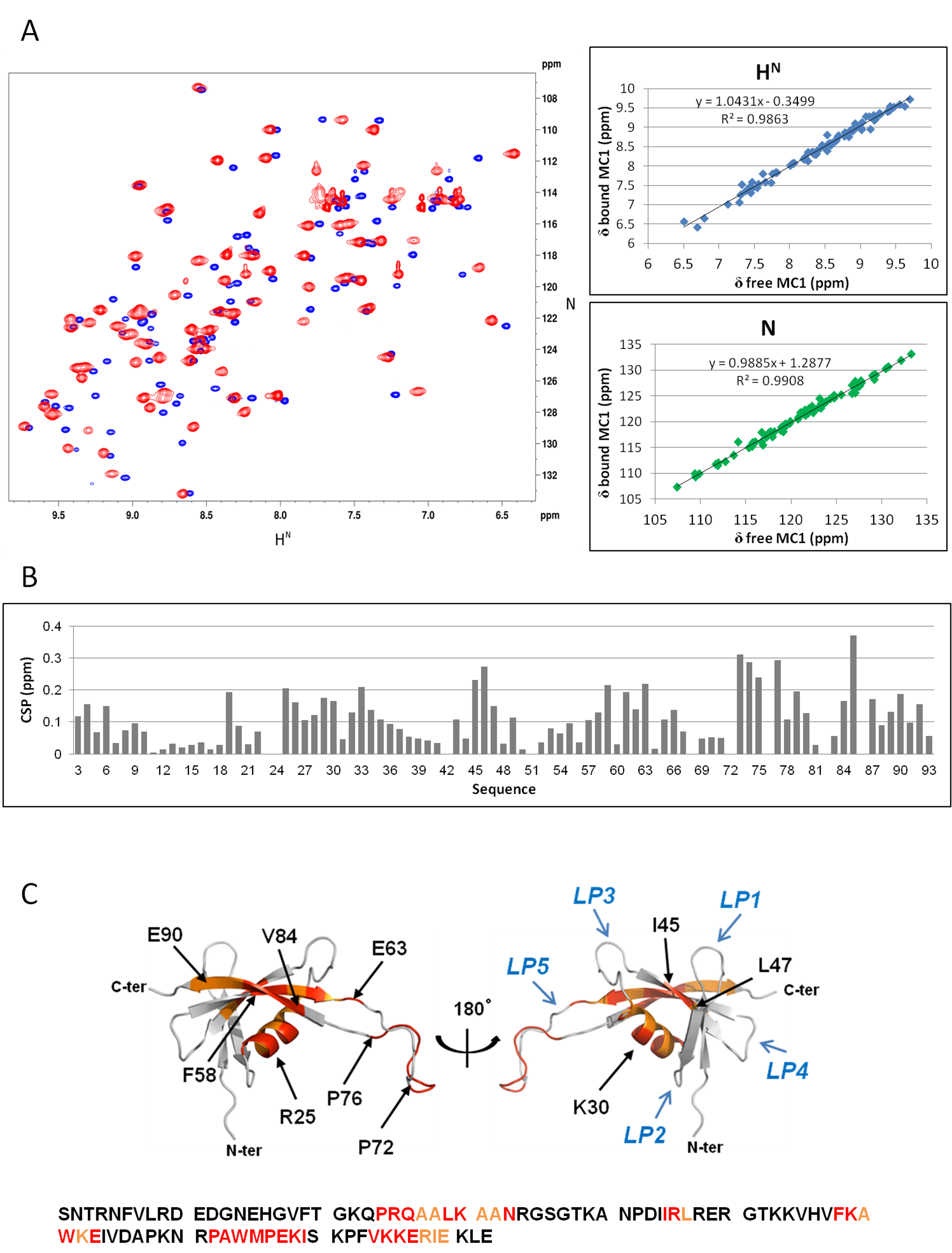

Supplement: Figure S5 — Analysis of MC1 CSP upon DNA-binding. (A) Superimposition of the 15N HSQC spectra of the free protein (blue) and the bound protein (red); HN and N chemical shifts as a function of free MC1 chemical shifts. A line was added to highlight the linear trend. (B) CSP values as a function of residue number. (C) Analysis of CSP with SAMPLEX: the MC1 protein backbone is shown as a cartoon; colored regions correspond to residues showing no (grey), medium (orange) or strong (red) chemical shift variations upon complexation of oligonucleotide to protein as recorded in NMR experiments; the five loops and key residues are labeled and the MC1 sequence with CSP is reported. Residues are located in five sites: Pro24-Arg34 (α-Helix), Ile45-Leu47 (β3-strand), Phe58-Glu63 (β4-strand), Pro72-Pro76 (loop LP5) and Val 84-Glu90 (β5-strand). (TIF) [file pone.0088809.s005.tif]

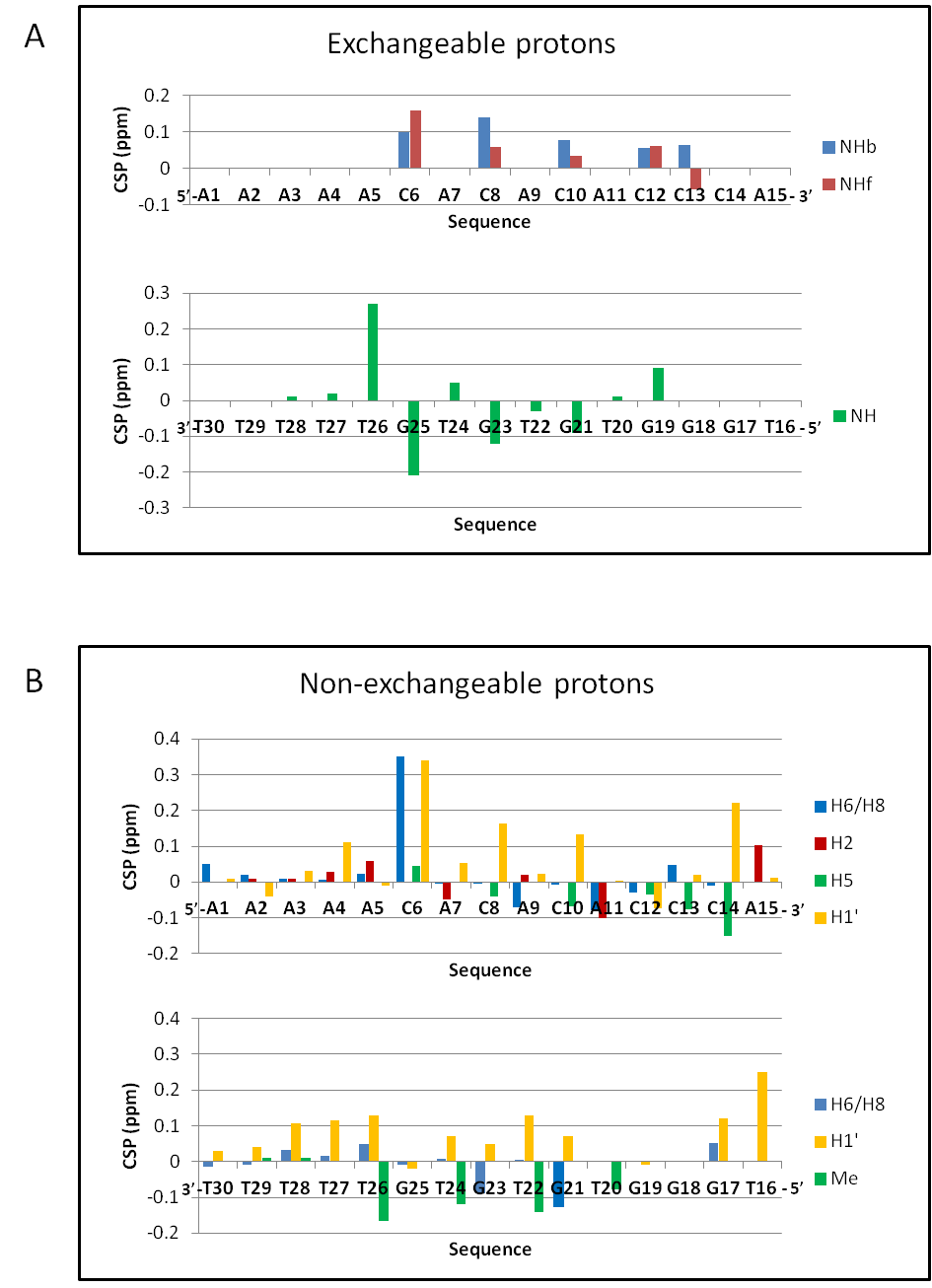

Supplement: Figure S6 — CSP analysis of DNA resonances upon MC1-binding. (A) CSP of the exchangeable protons upon MC1-binding: the bound amino protons (NHb), the free amino protons (NHf) and the imino protons (NH) are indicators of the Watson-Crick base pairing. (B) CSP of the nonexchangeable protons after MC1-binding. The anomeric H1' protons of deoxyribose and the aromatic H2 protons of adenine are located in the DNA minor groove. The H6 protons of cytosine/thymine, the H8 protons of adenine/guanine, the H5 protons of cytosine and the methyl group of thymine are located in the DNA major groove. (TIF) [file pone.0088809.s006.tif]

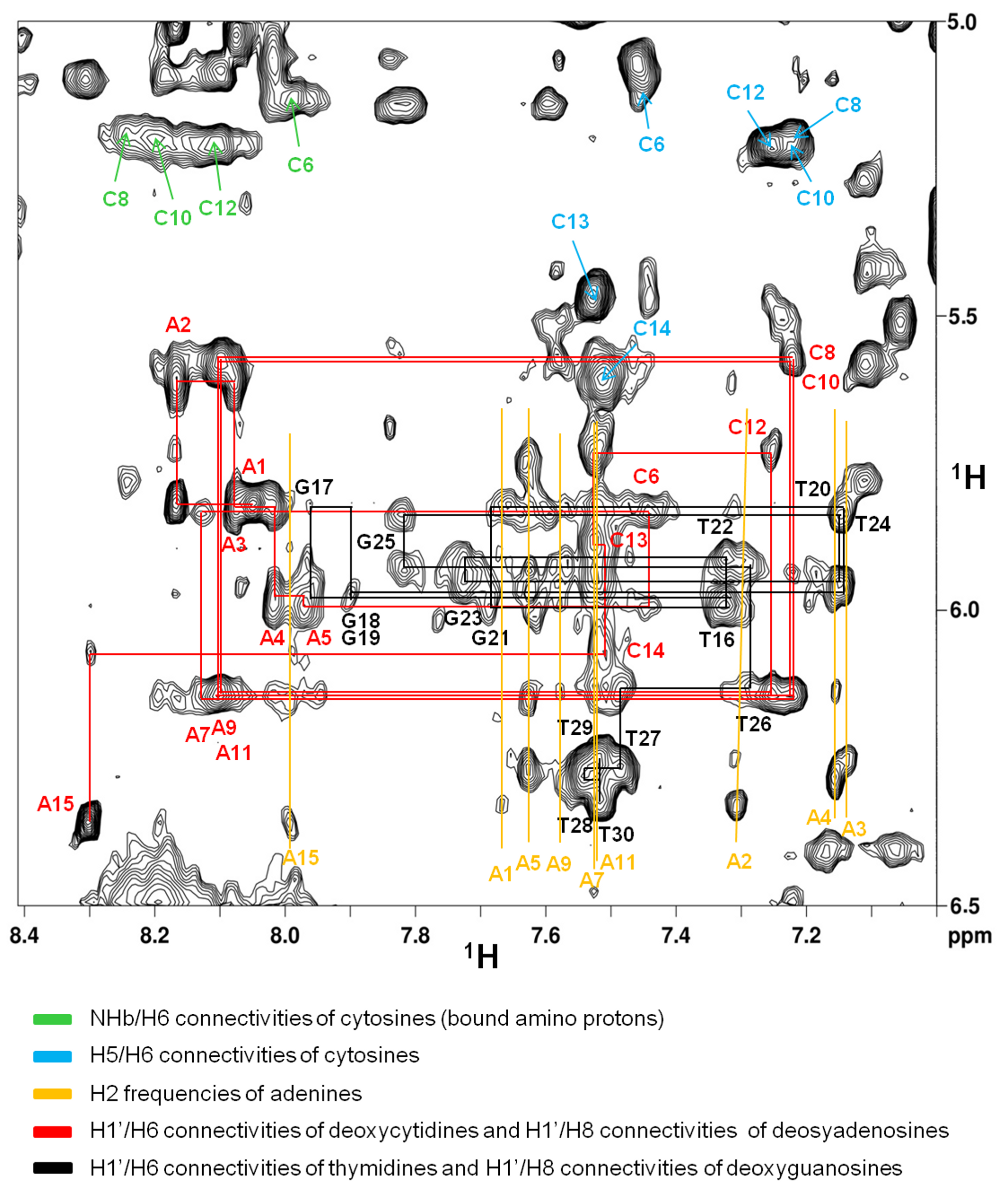

Supplement: Figure S7 — Assignment of the 15 bp DNA protons after binding to MC1. The NOESY spectrum was recorded on the complex (1 mM) in 10 mM phosphate buffer pH 6, 100 mM NaCl, 1 mM EDTA, 10% D2O at 26°C with a mixing time of 120 ms on a 600 MHz spectrometer. (TIF) [file pone.0088809.s007.tif]

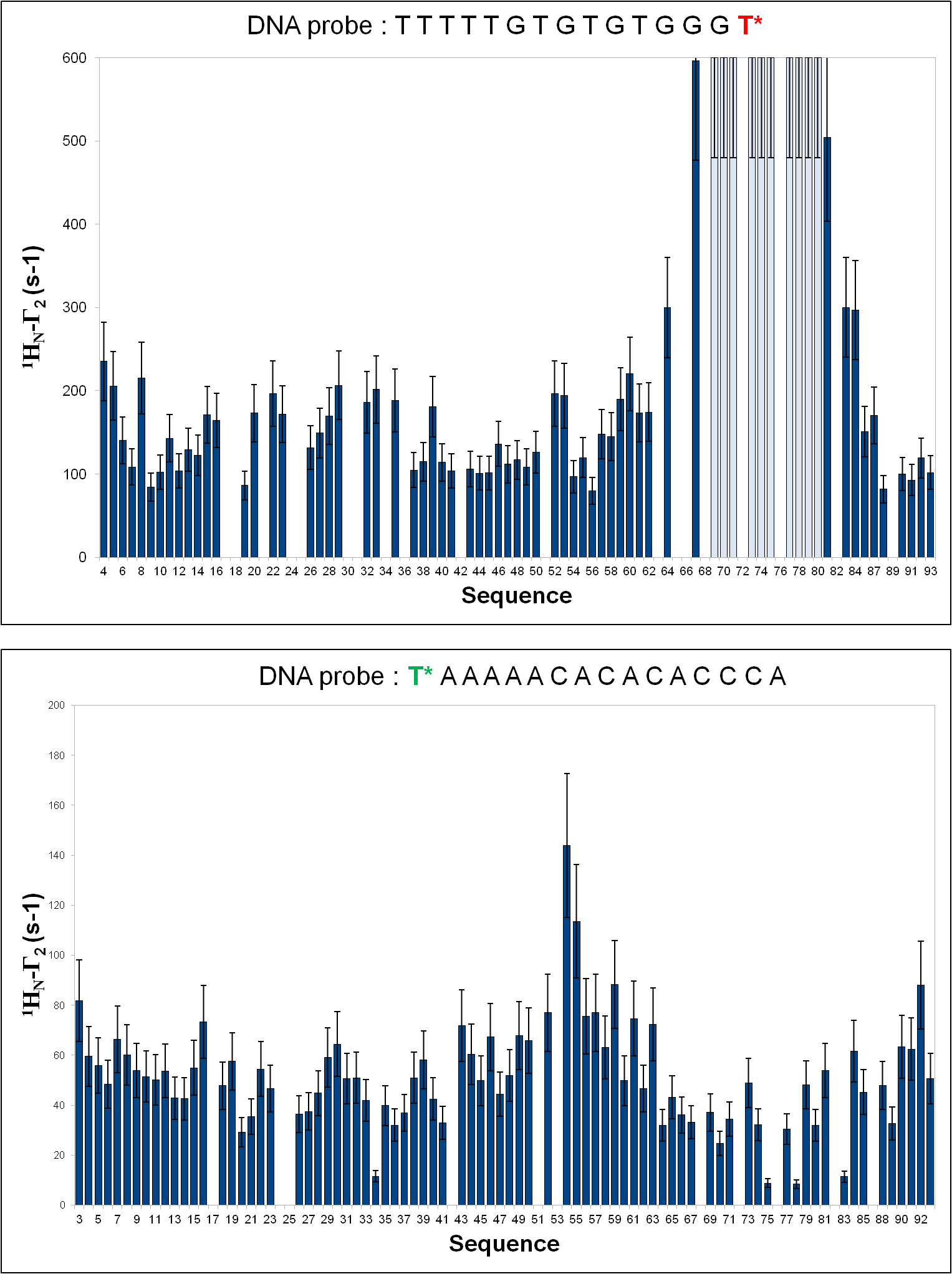

Supplement: Figure S8 — Intermolecular PREs for the MC1/DNA complex. Intermolecular PRE 1HN-Γ2 profiles obtained for each EDTA-Mn2+ DNA. (TIF) [file pone.0088809.s008.tif]

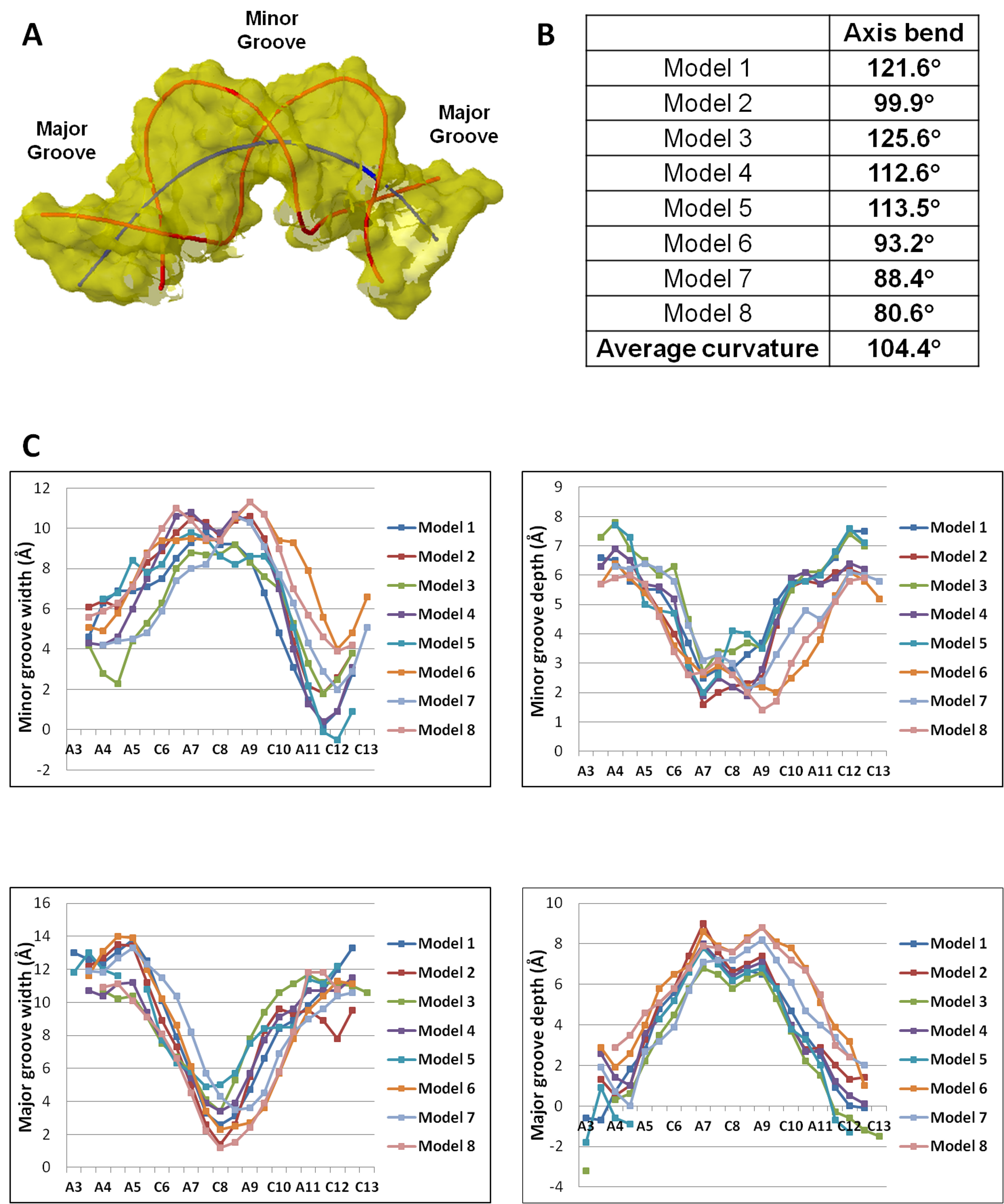

Supplement: Figure S9 — DNA groove geometry in the complex. The 8 models of 15 bp DNA bound to MC1 were analyzed using Curves+: (A) Schematic view of the bound DNA in the model 1 of the complex; the global helical axis is represented in blue and the backbone spines in red. (B) The DNA axis total bend, calculated for each model by Curves+, corresponds to the cumulative axis bend all along the oligonucleotide. (C) Groove parameters (width and depth) were calculated for each model. (TIF) [file pone.0088809.s009.tif]

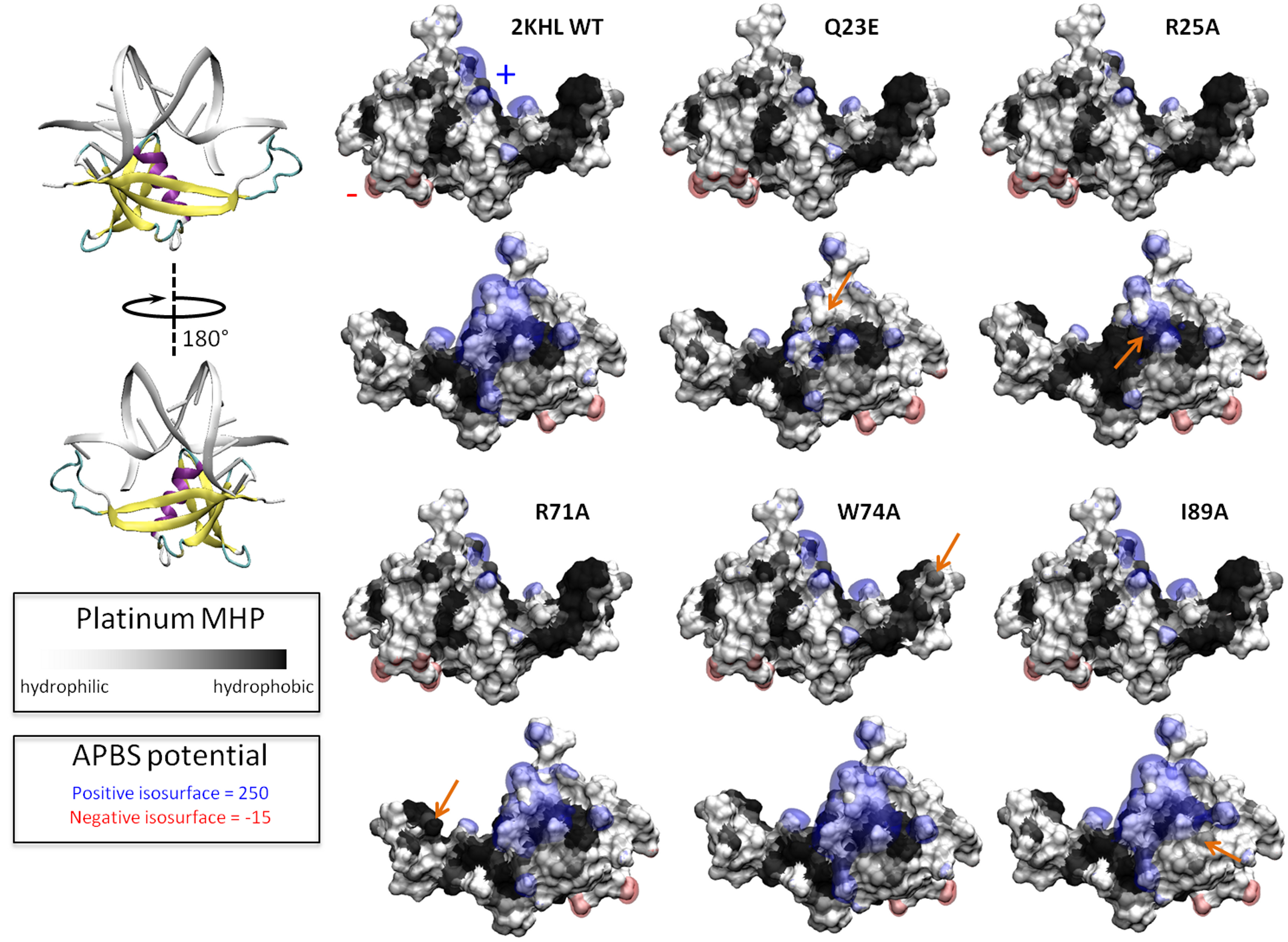

Supplement: Figure S10 — Three-dimensional representation of the surface potentials of the WT MC1 protein and its different mutants. Positive and negative electrostatic potential isosurfaces are respectively shown in blue (+250 kTe) and red (−15 kTe). Hydrophobicity/hydrophilicity is mapped on black/light grey molecular surfaces. Each mutation is located on the molecular surface of MC1with an arrow. (TIF) [file pone.0088809.s010.tif]
